# Breast cancer learning health system: Patient information from a data and analytics platform characterizes care provided

**DOI:** 10.1002/lrh2.10409

**Published:** 2024-02-13

**Authors:** Mark N. Levine, Joel Kemppainen, Morgan Rosenberg, Christopher Pettengell, Jessica Bogach, Tim Whelan, Ashirbani Saha, Jonathan Ranisau, Jeremy Petch

**Affiliations:** ^1^ Department of Oncology McMaster University Hamilton Ontario Canada; ^2^ Escarpment Cancer Research Institute Hamilton Ontario Canada; ^3^ Centre for Data Science and Digital Health Hamilton Health Sciences Hamilton Ontario Canada; ^4^ Pentavere Research Group Toronto Ontario Canada; ^5^ Department of Surgery McMaster University Hamilton Ontario Canada; ^6^ Department of Medicine McMaster University Hamilton Ontario Canada; ^7^ Population Health Research Institute, Hamilton Health Sciences Hamilton Ontario Canada; ^8^ Institute of Health Policy, Management and Evaluation University of Toronto Hamilton Ontario Canada

**Keywords:** breast cancer learning health system, clinical journey

## Abstract

**Purpose:**

In a learning health system (LHS), data gathered from clinical practice informs care and scientific investigation. To demonstrate how a novel data and analytics platform can enable an LHS at a regional cancer center by characterizing the care provided to breast cancer patients.

**Methods:**

Socioeconomic information, tumor characteristics, treatments and outcomes were extracted from the platform and combined to characterize the patient population and their clinical course. Oncologists were asked to identify examples where clinical practice guidelines (CPGs) or policy changes had varying impacts on practice. These constructs were evaluated by extracting the corresponding data.

**Results:**

Breast cancer patients (5768) seen at the Juravinski Cancer Centre between January 2014 and June 2022 were included. The average age was 62.5 years. The commonest histology was invasive ductal carcinoma (74.6%); 77% were estrogen receptor‐positive and 15.5% were HER2 Neu positive. Breast‐conserving surgery (BCS) occurred in 56%. For the 4294 patients who received systemic therapy, the initial indications were adjuvant (3096), neoadjuvant (828) and palliative (370). Metastases occurred in 531 patients and 495 patients died. Lowest‐income patients had a higher mortality rate. For the adoption of CPGs, the uptake for adjuvant bisphosphonate was very low, 8% as predicted, compared to 64% for pertuzumab, a HER2 targeted agent and 40.2% for CD4/6 inhibitors in metastases. During COVID‐19, the provincial cancer agency issued a policy to shorten the duration of radiation after BCS. There was a significant reduction in the average number of fractions to the breast by five fractions.

**Conclusion:**

Our platform characterized care and the clinical course of breast cancer patients. Practice changes in response to regulatory developments and policy changes were measured. Establishing a data platform is important for an LHS. The next step is for the data to feedback and change practice, that is, close the loop.

## 1 INTRODUCTION

Data generated through day‐to‐day clinical practice holds valuable clinical insight into the impact of treatment and care delivery on patient outcomes. This was recognized by the Institute of Medicine (IOM) through the learning health system (LHS); a continuous cycle or feedback loop in which scientific evidence informs clinical practice, while data gathered from clinical practice and administrative sources informs care and scientific investigation.^1^ In practice, the latter part of the loop is often missing because patient data are frequently siloed and/or unstructured, making it unsuitable for analysis with conventional statistical techniques. While manual retrospective chart reviews can allow such data to be used for research and quality improvement, they are time‐consuming and are limited by the scale of data that can be captured. The IOM recognized that having powerful, nimble and secure digital infrastructure is key to supporting an LHS.^2^

By leveraging routinely collected electronic data on patient care, an LHS can inform patient/oncologist decision‐making and institutional policy. Examples of questions that are important to a breast cancer LHS are whether clinical practice is consistent with guidelines, how practice changes in response to emerging evidence, how social determinants of health influence patient outcomes, and how patients are managed in scenarios where randomized trial data are not available.

The electronic health record (EHR) contains an abundance of patient information.^3^ However, harnessing clinical data from the EHR and making it available for research and quality assurance is a daunting challenge.^4^

The aim of our research is to create a breast cancer LHS at the Juravinski Cancer Center (JCC), a regional cancer center affiliated with McMaster University in Hamilton, Ontario. The JCC is the hub of cancer treatment services in south central Ontario, serving a catchment area of approximately 2.5 million people. Along with three affiliated community oncology clinics, it delivers all the systemic cancer therapy in the region and most of the radiation therapy.

In a previous pilot project, we demonstrated that the clinical course of 50 patients with locally advanced breast cancer could be characterized using a form of artificial intelligence called Natural Language Processing (NLP) applied to the clinical notes stored in the hospital's EHR.^5^ Building upon the work in the pilot study, we scaled up to a breast cancer data and analytics platform that automatically extracts and integrates data from our hospital's information systems and clinical documentation on a nightly basis.^6^ DARWEN, a commercially available medical NLP engine, was used to extract structured data from unstructured clinical documentation.^6^ In the current report, we demonstrate how this platform can characterize clinical care by building a comprehensive view of the patient journey and we describe opportunities provided by the platform to conduct research and quality improvement that define an LHS.

## 2 METHODS

This study was approved by the Hamilton Integrated Research Ethics Board (Project Number 15338‐C).

### 2.1 Data source

Our data platform currently includes 141 data elements prioritized by our clinicians.^6^ The data elements span many topic areas including demographics, patient encounters, pathology, medical imaging, radiation treatment, systemic therapy information, quality of life, recurrence, death and social determinants of health.

### 2.2 Patients

The cohort for the current report was assembled from 7019 new breast cancer patients seen at the JCC between 1 January 2014 and 3 June 2022. To be included in the cohort, histology had to be available. A total of 5986 (85.3%) patients had histology reported; invasive ductal or invasive lobular (4992), invasive mixed (169), ductal carcinoma in situ (DCIS) or lobular carcinoma in situ (LCIS) (677) and other (148). Of the 1033 patients with missing histology, almost half were missing because they had been referred to the JCC only for genetics assessment, pain control or social work consultation where such information is not required for referral. Patients with invasive ductal, invasive lobular or invasive mixed histology who did not have any biomarker information (hormone receptors, HER 2 Neu) were then excluded (118). Thus, the final study cohort consisted of 5868 patients.

### 2.3 Interventions

**FIGURE 1:**
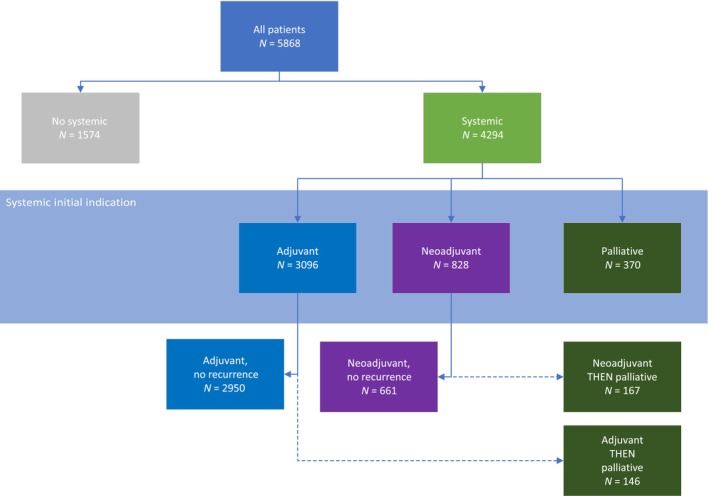
Systemic therapy attribution. Patients received systemic therapy as adjuvant therapy, neoadjuvant therapy or palliative treatment. Palliative indication based on patients who presented de novo with metastatic breast cancer plus adjuvant and neoadjuvant patients who developed metastatic disease.

Systemic therapy included chemotherapy, endocrine therapy and molecular targeted agents. The systemic therapy regimens were coded by the JCC pharmacy as adjuvant, neoadjuvant or palliative (Figure 1).

### 2.4 Outcomes

Mortality, including death from any cause, is reported along with the occurrence of a patient's first metastasis and site of metastases. The Edmonton Symptom Assessment Scale (ESAS), which is patient‐reported, is routinely collected at each clinic visit.^7^ The effect of adjuvant chemotherapy on patients is reported for several questions in ESAS before treatment, at mid‐course and after completion of adjuvant chemotherapy.

### 2.5 Social determinants of health

The importance of some social factors, for example, poverty and marginalization, on health has been recognized.^8^ Accordingly, we are building measurements of social determinants into the LHS. To explore the potential impact of the social determinants of health on our cohort we employed the Ontario Marginalization Index.^9^, ^10^ We considered four dimensions of marginalization (material deprivation, dependency, ethnic concentration and residential instability) and analysed the distribution of patients treated at the JCC based on quintiles of the Ontario population (quintile one being the least marginalized in Ontario and quintile five being the most marginalized). A more detailed description of this index and the dimensions of marginalization are provided in the supplement (Table S1).

### 2.6 Uptake of clinical practice guidelines and impact of policy changes

To evaluate the ability of our data platform to identify opportunities for research and quality improvement related to practice change, JCC medical oncologists specializing in breast cancer were asked to identify, based on their experience, two examples from the previous 10 years where a new clinical practice guideline (CPG) was published, and clinical practice changed immediately to be consistent with the CPG. These examples would be characterized as “game changers”. Example 1: In 2013, pertuzumab received regulatory approval for patients with metastatic HER 2 positive breast cancer.^11^ Example 2: In 2016, CD4/6 inhibitors received regulatory approval for the treatment of estrogen receptor‐positive metastatic breast cancer and their use was recommended through CPGs.^12^ The medical oncologists were also asked to provide an example from the previous 10 years where a CPG was published, but oncologists were reluctant to follow it, that is, it was less clear‐cut, and the response was mixed. Example 3: In 2017, guidelines recommended that bisphosphonates which block bone resorption be used in the adjuvant setting to prevent breast cancer recurrence and thus improve survival in post‐menopausal women.^13^

Since radiation therapy practice in Ontario is tightly coupled to policy guidance from the provincial cancer authority, Cancer Care Ontario (CCO) radiation oncologists were asked to identify a recent provincial policy change that may have had a significant impact on practice. Example 4: On 25 April 2020, in response to the COVID‐19 pandemic, CCO recommended decreasing the number of fractions for postoperative breast irradiation.^14^

### 2.7 Analysis

Descriptive statistics were used to analyze the patient population and the care provided.

Practice change in radiation therapy was evaluated using an interrupted time series design, a robust quasi‐experimental method for evaluating the impact of quality improvement initiatives and large‐scale health interventions when randomization is not possible.^15^ We employed an Autoregressive Integrated Moving Average (ARIMA) model for this analysis to account for seasonality and autocorrelation.^16^ Monthly values for mean fractions per patient were calculated by attributing all fractions provided to patients to the month in which their radiation therapy was initiated.

Analyses were carried out in Python (version 3.9.6) using the following packages: matplotlib (3.5.3), pandas (1.4.2), seaborn (0.11.2) and stats (0.13.2) models.

## 3 RESULTS

### 3.1 Patients

Our platform proved effective in allowing us to rapidly characterize the patients we care for (Table 1). The average age is 62.5 (SD 13.2) years. Invasive ductal carcinoma is the most common histology (78.0%) with invasive lobular next in frequency (84.5%). A total of 606 (11.2%) patients presented with DCIS only. Metaplastic cancers, squamous cell cancers and Phyloides tumors are in the “Other” histology category. A total of 77% of tumors were ER‐positive, 13.0% ER‐negative and 15.5% HER 2 Neu positive. A total of 56% of patients underwent BCS. Approximately half the patients had hypertension and one quarter had diabetes.

**TABLE 1:** Baseline characteristics.

Characteristic	Result *N* = 5868
Mean age (SD) years	62.5 (13.2)
Sex	
Female	5832
Male	36
Tumor histology^a^	
Invasive ductal	4579
Invasive lobular	496
Invasive mixed	168
Other	148
DCIS alone	660
LCIS alone	17
Biomarker	
ER (+/−/unknown)	4535/763/570
PR (+/−/unknown)	3574/1417/877
Her2 neu (+/−/indeterminant/unknown)	910/3844/19/1095
Triple‐negative	431
Surgery	
Breast conservation	3307
Modified radical mastectomy	794
Mastectomy	1500
Axillary dissection	2125
Sentinel node biopsy	2246
Comorbidities	
Atrial fibrillation	478
Stroke	317
Hypertension	2642
Chronic obstructive pulmonary disease	368
Coronary artery disease	465
Diabetes	1160
Edmonton Symptom Assessment Score (no. with baseline)	3616

^a^ Histology can be counted more than once for a patient.

### 3.2 Interventions

Using our data platform, we determined that 4294 (73.2%) of our patients received systemic therapy; 3096 were for the initial indication of adjuvant (given after surgery), 828 neoadjuvant (prior to surgery) and 370 palliative treatments (Figure 1).

Adjuvant chemotherapy was administered to 1206 patients. The most common adjuvant chemotherapy regimen administered was dose dense Adriamycin, Cyclophosphamide, Taxol (DDACT; *n* = 934, 77.4%) (Table S2 in supplement), followed by Adriamycin/Cyclophosphamide (AC; *n* = 122) and Taxotere/Cyclophosphamide (TC; *n* = 108). A total of 576 (18.5%) patients also received adjuvant trastuzumab. Adjuvant endocrine therapy was administered to 2731 patients: 1890 patients received adjuvant endocrine therapy alone and 841 received endocrine therapy with chemotherapy. The frequency of use of endocrine agents is shown in Table S2; 2037 patients (74.6%) received anastrozole, 1211 (44.3%) tamoxifen, 646 (23.7%) letrozole and 126 (4.6%) exemestane.

The most common chemotherapy regimen administered in neoadjuvant patients was DDACT (*n* = 676) (Table S2). A total of 169 patients in this group also received trastuzumab.

The most frequent first‐line chemotherapy regimens used in patients with metastatic breast cancer were capecitabine (190 patients), weekly doxorubicin (33 patients) and weekly paclitaxel (141) (Table S1). For the HER2 Neu‐positive patients, 111 patients received trastuzumab and pertuzumab and 25 patients received trastuzumab emtansine (Table S2). Endocrine therapy (anastrozole, letrozole, exemestane, tamoxifen or fulvestrant) was used in 491 patients with metastatic breast cancer (palliative indication) (Table S2).

We were also able to determine that of the 3307 patients who underwent BCS, 2549 patients (77.1%) received postoperative breast irradiation. The average number of fractions of radiation delivered was 16 (ranges 1–39).

### 3.3 Outcomes

The degree of follow‐up captured by our data platform is shown in Figure S1. The proportion of patients with <2 years of follow‐up is quite constant from 2014 to 2019 (mean 10%), with an expected increase in 2020 and 2021 (since two full years have not passed for many of these patients). Similarly, the further back in time we look we observe an expected increase in the proportion of patients who have died.

Five hundred thirty‐one (9.0%) patients experienced metastasis. This includes adjuvant, neoadjuvant and no systemic therapy patients in whom metastases subsequently occurred and patients who presented de‐novo with metastases. The most common sites of metastases were bone, liver, lung and brain. The number of patients who were confirmed to have died was 495.

The patient reported symptoms related to adjuvant chemotherapy using ESAS before treatment, at mid‐course and after completion of adjuvant chemotherapy as shown in Figure S2 in the supplement. Results for four questions are shown. Tiredness and pain increased, while anxiety dropped and there was no change in nausea.

### 3.4 Social determinants of health

**FIGURE 2:**
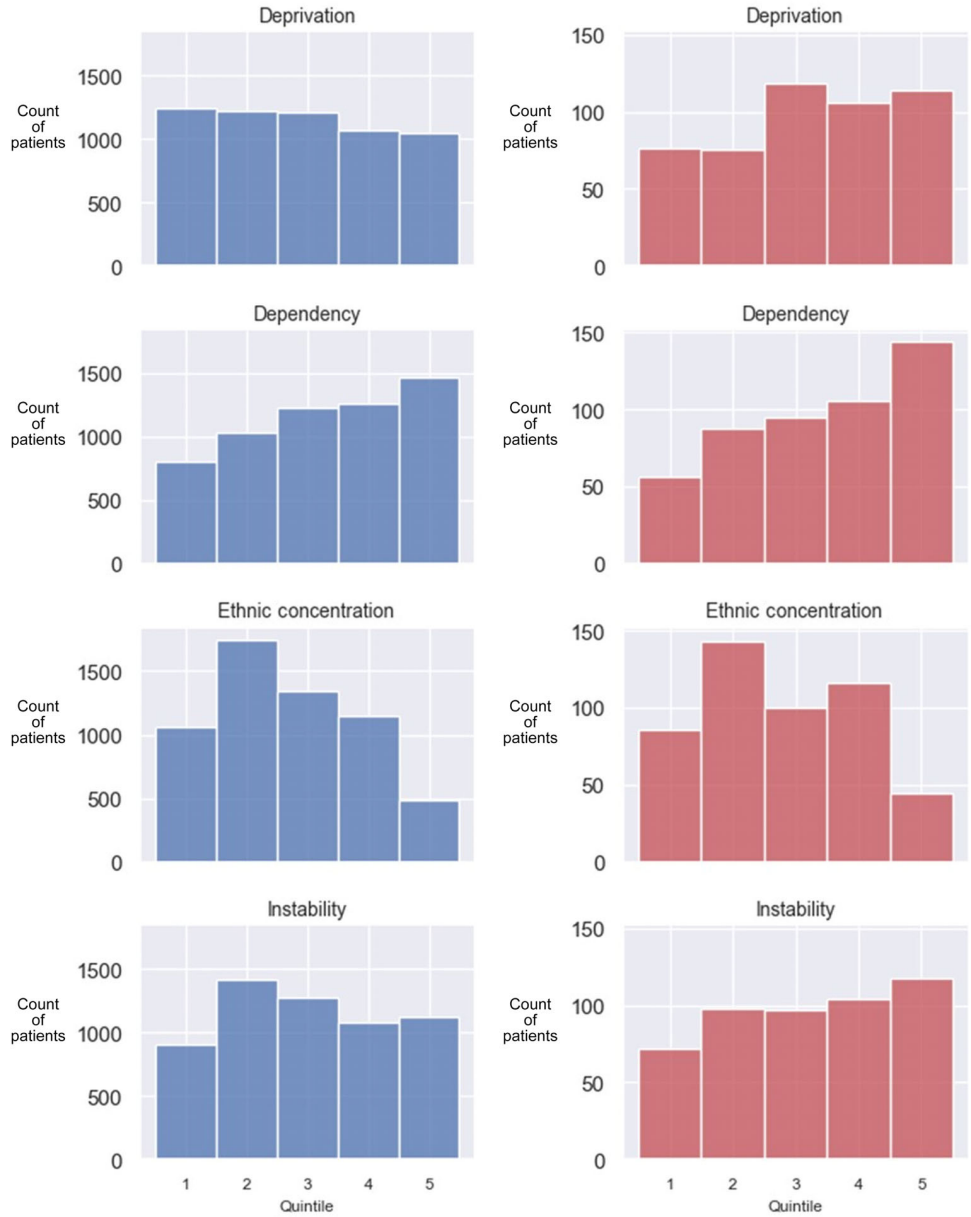
Baseline socioeconomic characteristics of the entire cohort. Each plot describes a dimension of marginalization with population quintiles along the x‐axis and the count of patients in each quintile along the y‐axis. Plots in blue are for all patients, while plots are in red for those patients who have died. Quintiles are assigned to patients based on the dissemination area in which they live (dissemination areas are the smallest special areas measured by the Canadian census—they are relatively stable geographic units with an average population of 400–700 persons). Quintiles range from one (least marginalized) to five (most marginalized). Thus, patients in quintile five on the marginalization scale reside in one of the most deprived 20% of areas in Ontario. The deprivation dimension is closely connected to poverty.

The baseline socioeconomic characteristics of the cohort are described in Figure 2. There is a relatively even distribution of patients with respect to material deprivation, which is closely related to poverty, with slightly fewer patients from the most deprived quintile. Conversely, there was a higher proportion of patients from the most deprived quintiles who have died.

### 3.5 Uptake of clinical practice guidelines and impact of policy changes

**FIGURE 3:**
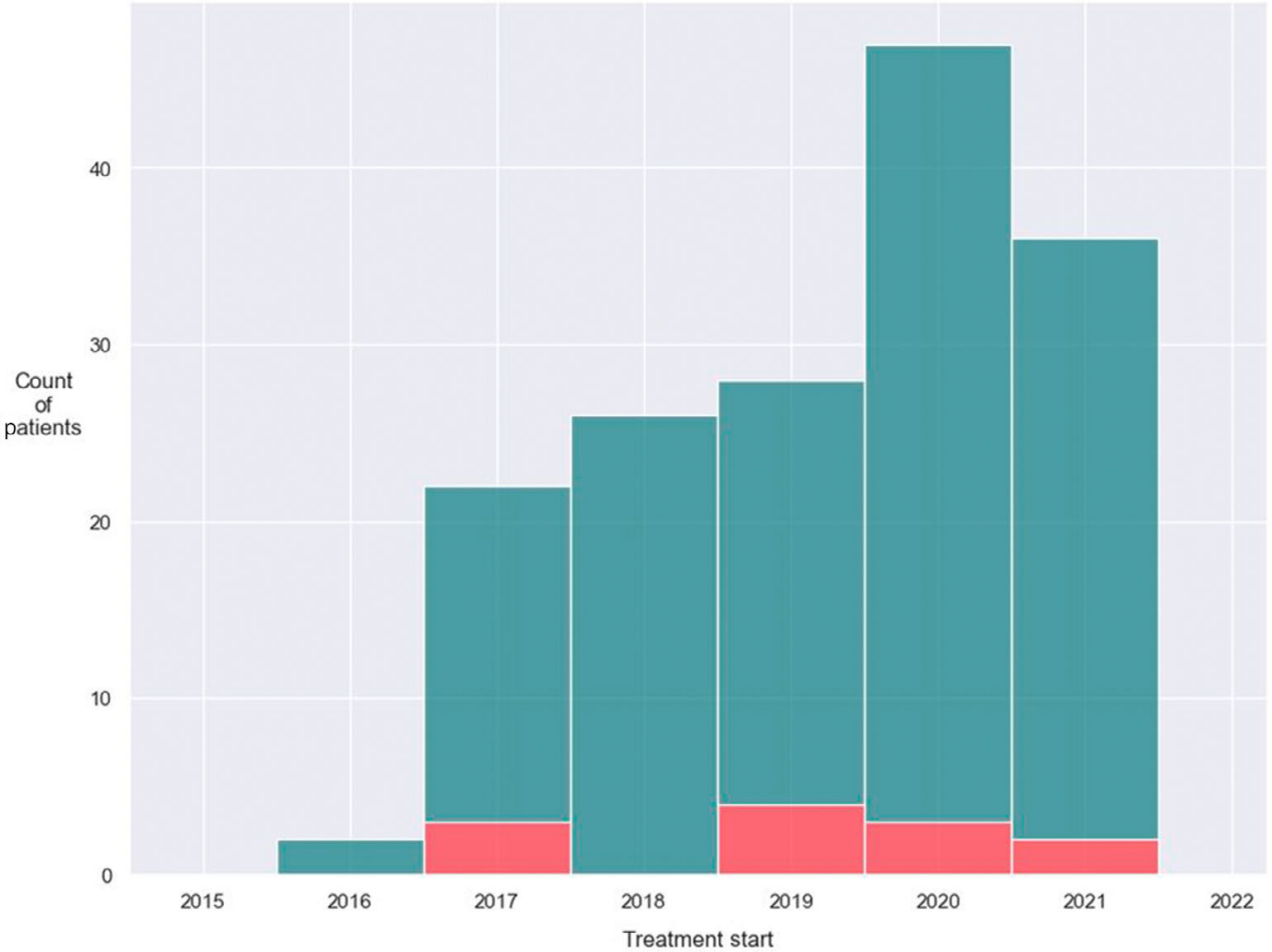
Introduction of CD4/6 Inhibitors. Number of patients starting a CD4/CD6 inhibitor over a 12‐month period. Green = Palbociclib and Red = Ribociclib.

**FIGURE 4:**
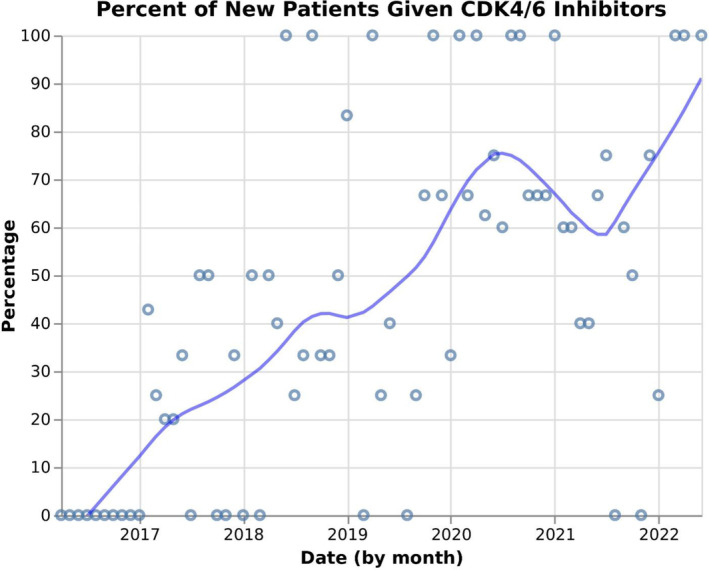
Introduction of CD4/6 Inhibitors—(LOESS) Plot. A locally estimated scatterplot smoothing (LOESS) plot is a nonparametric method for smoothing a series of data (Cleveland, W.S., 1979: Robust locally weighted regression and smoothing scatterplots. Journal of the American Statistical Association, 74, 829–836).

The adoption of pertuzumab was identified as an example of rapid uptake of a guideline by the JCC medical oncologists (example 1). A total of 72 (64.3%) of 112 patients with metastatic HER2‐positive disease received pertuzumab as a part of their antineoplastic systemic regimen. The second example of rapid uptake identified a priori was CD4/6 inhibitors. Of 303 patients over 50 years of age with ER‐positive metastatic breast cancer, 122 (40.2%) took a CD4/6 inhibitor. Their adoption into practice is shown in Figure 3. To further examine the uptake, a locally estimated scatterplot smoothing (LOESS) plot was performed on the scatter plot data (Figure 4). The medical oncologists postulated that there would be poor uptake of the use of adjuvant bisphosphonates (example 3). Of 2264 patients over 50 years of age with breast cancer who received systemic adjuvant therapy after 1 January 2017, only 180 (8.0%) received adjuvant bisphosphonate.

**FIGURE 5:**
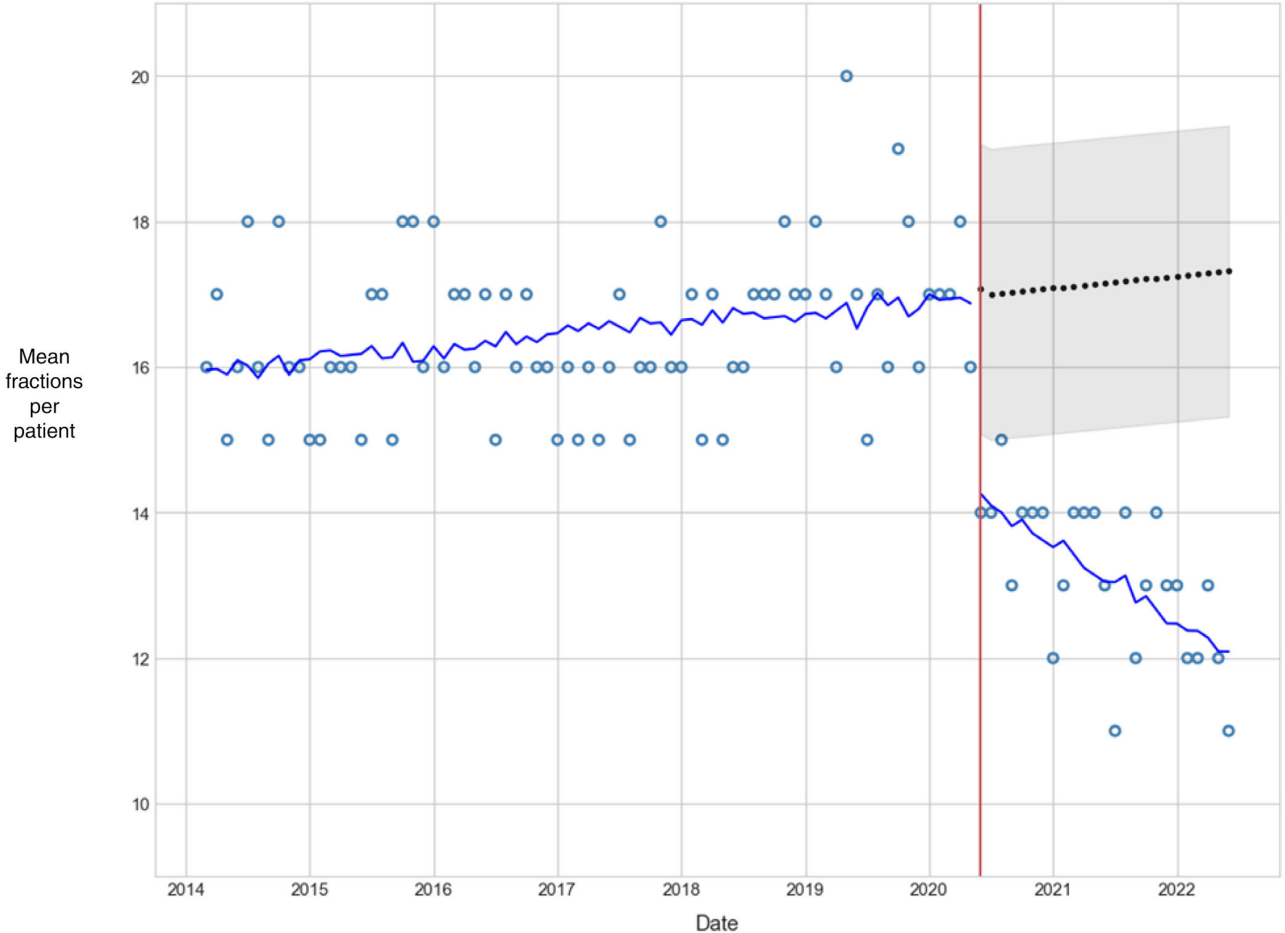
Interrupted time series analysis of mean fractions per patient per month following lumpectomy surgery using autoregressive integrated moving average (ARIMA). A total of 2549 patients received postoperative radiotherapy following lumpectomy. Blue points indicate the mean fractions provided post‐lumpectomy to patients each month. The blue line is an ARIMA time series model fit to the actual observations. The red line indicates the date (25 April 2020) when guidance was introduced by Cancer Care Ontario (CCO) to provide 15/16 fractions to most lumpectomy patients and to consider five fractions (ultra‐hypofractionation) for low‐risk patients, rather than the previous guidance of 15–25 fractions. The black dotted line is the forecast ARIMA time series which represents the counterfactual projection of what would have been expected to occur had no guideline change been implemented. The gray shaded area represents the 95% confidence interval of the time series forecast. This analysis suggests that mean fractions per patient have been increasing slightly since 2014, and that the new guidance introduced by CCO in April 2020 was responsible for a significant reduction and that uptake of the new guidance increased throughout 2021 and 2022.

In response to COVID‐19, CCO recommended decreasing the number of fractions for postoperative breast irradiation.^13^ As a result of the CCO recommendation (example 4), there was a marked reduction in the average number of fractions, from 17 prior to the pandemic to 12 from May 2020 onward (Figure 5).

## 4 DISCUSSION

The overall goal of our research was to describe the care provided to breast cancer patients at our regional cancer center using information from a novel data and analytics platform.^6^ We have shown that individual patient data on patient factors, for example, comorbid medical conditions, social determinants, tumor characteristics (e.g., hormone receptors), HER2 Neu expression, diagnosis, treatments and follow‐up outcomes can be extracted from this platform and assembled to characterize the care patients receive throughout their journey. Having such information is essential to the creation of an LHS. It can be used both to identify areas for quality improvement and research, as well as to monitor the impact of change efforts related to new clinical guidelines and policies.

Increased use of granular neighborhood‐level socioeconomic data has been recently highlighted as an important opportunity for cancer research.^17^, ^18^, ^19^ Our data reveals that while our cancer center serves a relatively even distribution of the population with respect to material deprivation, survivorship for breast cancer appears to be much better for the rich than for the poor. The same is true for housing instability; patients from stable neighborhoods appear to be less likely to die than patients from neighborhoods with high instability. These are interesting observations coming from a system with universal healthcare access. We did not analyze whether the poorest patients or those with the most unstable housing received different treatments than the other patient groups. This is for future work.

An important function of a data platform such as ours is to measure uptake of CPGs in routine clinical practice. The validity of our data platform was established by assessing the degree with which the data on treatments received agreed with theoretical constructs. A priori, medical oncologists from the center's breast cancer disease site team identified two significant practice changes that were reflected in guidelines and would be manifest in our analyses. They also identified a scenario where they predicted uptake would be low. The uptake of pertuzumab, a recombinant humanized monoclonal antibody that binds to a separate antigenic region of the HER2 extracellular domain than trastuzumab, was relatively high as predicted a priori by the medical oncologists.^11^ Approximately one‐third of patients with HER2‐positive metastatic disease did not receive pertuzumab. Possible explanations are the presence of cardiac disease, decreased cardiac ejection fraction because of prior exposure to adjuvant trastuzumab, or patients who received pertuzumab at one of the community oncology sites and this data was not captured in the data platform. Our results show the uptake of CD4/6 inhibitors was high once approved by the government regulatory agency.^12^ It is not surprising that the use of palbociclib was much higher than ribociclib, likely because it was introduced first.^20^ However, we anticipate that soon our data will reflect that ribociclib use will overtake that of palbociclib because of the recently reported improvement in survival with ribociclib.^21^ The uptake of adjuvant bisphosphonate was low as predicted. The published CPG was controversial.^13^ Reasons for this were questions about the validity of the data, lack of clarity on their mechanism of actions, small magnitude of benefit, side effects and uncertainty about the logistics of therapy (time to start and dosing intervals).^13^, ^22^

Post hoc consideration of the patient data provides an opportunity for us to speculate on the congruence of the data with preconceived notions. For example, the most common adjuvant or neoadjuvant chemotherapy regimen used was DDACT in keeping with CPGs.^23^ For metastatic disease, less toxic chemotherapy was chosen, for example, oral capecitabine, weekly doxorubicin or weekly paclitaxel. We speculate that this real‐world evidence supports the construct that a goal of treatment for metastases is to control the cancer and maintain quality of life for as long as possible.^24^

Another important function of a data platform is to measure changes in practice resulting from policy changes. The COVID‐19 pandemic resulted in an abrupt change in practice at the JCC and worldwide.^25^, ^26^ To reduce patient visits to the JCC there was a substantial change in radiation therapy to shorter radiation treatments. Although this was a pragmatic decision, it was based on guidelines from CCO^14^ and emerging data on ultra‐hypofractionation.^27^ Our platform clearly captured the change over time, a valuable ability for a rapid LHS that needs to monitor the impact of its change initiatives.

The routine use of patient‐reported outcome measures for clinical decision‐making in oncology clinical practice is being advocated.^28^ In 2007, CCO encouraged all their regional cancer centers to routinely collect information on symptoms at each clinic visit with the ESAS (patient self‐reported).^29^, ^30^ The pattern of ESAS scores in patients receiving adjuvant chemotherapy was consistent with expectations. Patients had high anxiety levels on their first visit to the clinic. We speculate that they are waiting for information on their treatment and prognosis. Once there is a plan, anxiety drops considerably. Pain increased over time and after completion of treatment. This is likely related to the neuropathic pain from paclitaxel that can last for months after completion of chemotherapy. These two examples are encouraging and support that in our LHS, patient‐reported outcomes can be connected to other clinical data.

Administrative data sources such as SEER have been used to generate evidence from real‐world practice. However, these sources do not tend to enable cancer centers to leverage their own data to monitor and improve care in their regions. The potential of the EHR to be a valuable resource for regional research and quality improvement in cancer has been touted for a long time.^3^, ^31^ Progress has been slow, due in large part to the siloed and unstructured nature of clinical documentation. The Oncoshare project has linked multiple local and national breast cancer data sources to enable population‐based^32^ and NLP research topics.^33^ The potential for integrated data to enable LHS activities was recently described by Hernandez‐Boussard, who combined data from Stanford University EHR, the California Cancer Registry and the Veteran's Health Administration to characterize care provided to their patients.^34^ Recently, Morin and colleagues described the creation of an information technology infrastructure using data from cancer patients at the University of California San Francisco^35^ to generate prognostic models in breast cancer using NLP. In our study, we extracted data from a platform to describe the clinical course of and care provided to breast cancer patients at a regional cancer center from diagnosis to last follow‐up or death. To the best of our knowledge, this is the first time this has been reported.

### 4.1 Limitations

Patients were not included in the study cohort if histology was missing. Many of the patients with missing histology were referred to the JCC only for genetics assessment, pain control or social work consultation, where such information is not required for referral. Typically, such patients had a consultation only. We do not feel that the loss of these patients has any material impact on the generalizability of the results. However, 118 patients were excluded because of the absence of all biomarkers. We did not compare the characteristics of these patients with the rest of the cohort. It is conceivable that they may have been older or had smaller tumors, potentially resulting in bias.

Breast cancer is usually staged clinically at presentation based on the TNM system; T = size, N = axillary nodes and M = metastases.^36^ Stage correlates with prognosis. Sometimes staging is performed by the physician or trained clerical abstractors. Accurate staging of cancer can be a challenge.^37^ We collected data on tumor size, presence of nodes and presence of metastases separately, but chose not to aggregate these data into a stage for simplicity. We determined that having the tumor data in this granular format would not hinder our ability to mine the data for prognosis. Staging is important in the field of oncology. The individual TNM components exist in our platform and over the next year we will be improving the platform by assembling them into stages.

Because some patients receive follow‐up care through their family physician and/or at a community hospital, recurrences and deaths may very well be underreported. Linking with a provincial death registry will address this.

Our platform has been built for the information technology (IT) system at Hamilton Health Sciences. There will be costs to adapt the IT systems in other hospitals to our platform. The cost and feasibility will be influenced by the compatibility of EHRs.

Collecting and evaluating patient data based on real‐world clinical practice is not like conducting a clinical trial where patient follow‐up visits and tests are scheduled at regularly specified times.^38^ Moreover, real‐world practice at a regional cancer center is shaped by system‐level factors that vary by jurisdiction which may limit the generalizability of some research using this data. However, our goal with this work is not to create generalizable knowledge, rather we are looking for system‐specific knowledge that can be continuously integrated into ongoing clinical practice.

The establishment of an LHS is a complex multi‐step process. Although the creation of the data platform is an important step, we have much more work to do. Having the data platform stands us in good stead for future initiatives. However, closing the loop to improve practice can involve many factors. Examples include but are not limited to, extraction and analysis of the data, clinician trust of the data, need for additional data elements (e.g., reconstructive surgery) that are not in the platform and operational processes for feedback to clinicians. Changing behavior is a very complex process and is an important domain for study in our LHS.

## 5 CONCLUSION

We have demonstrated how a novel data and analytics platform can be used to characterize the care provided at a regional cancer center; from describing the patient population to quantifying the distribution of systemic therapies and the number of radiation treatments given and assessing outcomes such as symptoms, recurrence and mortality. Importantly, it can be used to measure practice changes in response to new clinical guidelines and policy changes. These analyses form the foundation of our efforts to create an LHS for breast cancer at our center.

## FUNDING INFORMATION

Grant‐in‐aid Roche Canada.

## CONFLICT OF INTEREST STATEMENT

Dr. Christopher Pettengell is an employee of PENTAVERE Inc.; this company provided the Darwen natural language processing (NLP) tool for the development of our learning health system platform. All other authors declare no conflict of interest.

## Supporting information


**Data S1.** Supporting information.

## References

[1] Institute of Medicine . The Learning Healthcare System: Workshop Summary. Washington, DC: The National Academies Press; 2007. doi:10.17226/11903 21452449

[2] Institute of Medicine . Digital Infrastructure for the Learning HealthSystem: The Foundation for Continuous Improvement in Health and Health Care: Workshop Series Summary. Washington, DC: The National Academies Press; 2011. doi:10.17226/12912 22379651

[3] Deasy JO , Stetson PD . A platform for continuous learning in oncology. Nature Cancer. 2021;2:675‐676. 35121947 10.1038/s43018-021-00239-z

[4] Honeyford K , Expert P , Mendelsohn EE , et al. Challenges and recommendations for high quality research using electronic health records. Front Digit Health. 2022;4: 940330. doi:10.3389/fdgth.2022.940330 36060540 PMC9437583

[5] Levine MN , Alexander G , Sathiyapalan A , Agrawal A , Pond G . Learning health system for breast cancer: pilot project experience. JCO Clin Cancer Inform. 2019;3:1‐11. doi:10.1200/CCI.19.00032 31369338

[6] Petch J , Kempainnen J , Pettengell C , et al. Developing a data and analytics platform to enable a breast cancer learning health system at a regional cancer center. JCO Clin Cancer Inform. 2023;7:e2200182. doi:10.1200/CCI.22.00182 37001040 PMC10281330

[7] Bruera E , Kuehn N , Miller MJ , Selmser P , Macmillan K . The Edmonton Symptom Assessment System (ESAS): a simple method for the assessment of palliative patients. J Palliat Care. 1991;7:6‐9. 1714502

[8] Rahul V , Reddy N , Giron N , et al. The social determinants of health—moving beyond screen‐and‐refer to intervention. N Engl J Med. 2023;389:569‐573. 37590456 10.1056/NEJMms2211450

[9] Matheson FI , Moloney G , van Ingen T . Ontario Agency for Health Protection and Promotion (Public Health Ontario). 2016 Ontario Marginalization Index: User Guide. 1st Revision. Toronto, ON: St. Michael's Hospital (Unity Health Toronto); 2022 Joint publication with Public Health Ontario.

[10] Khan AM , Urquia M , Kornas K , et al. Socioeconomic gradients in all‐cause, premature and avoidable mortality among immigrants and long‐term residents using linked death records in Ontario, Canada. J Epidemiol Community Health. 2017;71(7):625‐632. doi:10.1136/jech-2016-208525 28289039 PMC5485756

[11] Giordano SH , Temin S , Kirshner JJ , et al. Systemic therapy for patients with advanced human epidermal growth factor receptor2‐positive breast cancer: American Society of Clinical Oncology clinical practice guideline. J Clin Oncol. 2014;32:2078‐2099. 24799465 10.1200/JCO.2013.54.0948 PMC6076031

[12] Turner NC , Ro J , Andre F , et al. Palbociclib in hormone‐receptor‐positive advanced breast cancer. N Engl J Med. 2015;373:209‐219. 26030518 10.1056/NEJMoa1505270

[13] Dhesy‐Thind S , Fletcher GG , Blanchette PS , et al. Use of adjuvant bisphosphonates and or the bone‐modifying agents in breast cancer: a CCO and American Society of Clinical Oncology clinical practice guideline. J Clin Oncol. 2017;35:2062‐2081. 28618241 10.1200/JCO.2016.70.7257

[14] Ontario Health Cancer Care Ontario . COVID‐19 Supple11mental Clinical Guidance for Patients with Cancer #2: Management of Patients with Breast Cancer being considered for Radiation Therapy. 2020.

[15] Hategeka C , Ruton H , Karamouzian M , Lynd LD , Law MR . Use of interrupted time series methods in the evaluation of health system quality improvement interventions: a methodological systematic review. BMJ Glob Health. 2020;5(10):e003567. doi:10.1136/bmjgh-2020-003567 PMC7559052 33055094

[16] Schaffer AL , Dobbins TA , Pearson SA . Interrupted time series analysis using autoregressive integrated moving average (ARIMA) models: a guide for evaluating large‐scale health interventions. BMC Med Res Methodol. 2021;21:58. doi:10.1186/s12874-021-01235-8 33752604 PMC7986567

[17] Singh GK , Jemal A . Socioeconomic and racial/ethnic disparities in cancer mortality, incidence, and survival in the United States, 1950–2014: over six decades of changing patterns and widening inequalities. J Environ Public Health. 2017;2017:2819372. doi:10.1155/2017/2819372 28408935 PMC5376950

[18] Swords DS , Scaife CL . Granular neighborhood‐level socioeconomic data: An opportunity for a different kind of precision oncology? 2021. doi:10.1016/j.amjsurg.2021.01.022 33500142

[19] Mora J , Krepline AN , Aldakkak M , et al. Adjuvant therapy rates and overall survival in patients with localized pancreatic cancer from high Area Deprivation Index neighborhoods. Am J Surg. 2021;222(1):10‐17. doi:10.1016/j.amjsurg.2020.12.001 33308823

[20] Hortobagyi GN , Stemmer SM , Burris HA , et al. Ribociclib as first‐line therapy for HR‐positive, advanced breast cancer. N Engl J Med. 2016;375:1738‐1748. 27717303 10.1056/NEJMoa1609709

[21] Hortobagyi GN , Stemmer SM , Burris HA , et al. Overall survival with ribociclib plus letrozole in advanced breast cancer. N Engl J Med. 2022;386:942‐950. 35263519 10.1056/NEJMoa2114663

[22] Eisen A , Somerfield MR , Accordino MK , et al. Use of adjuvant bisphosphonates and other bone‐modifying agents in breast cancer: ASCO‐OH (CCO) guideline update. J Clin Oncol. 2022;40:787‐800. 35041467 10.1200/JCO.21.02647

[23] Moy B , Rumble BR , Carey LA , Chemotherapy and Targeted Therapy for HER2‐Negative Metastatic Breast Cancer that is Either Endocrine‐Pretreated or Hormone Receptor–Negative Expert Panel . Chemotherapy and targeted therapy for human epidermal growth factor receptor 2–negative metastatic breast cancer that is either endocrine‐pretreated or hormone receptor–negative: ASCO guideline rapid recommendation update. J Clin Oncol. 2022;40(26):3088‐3090. 35926153 10.1200/JCO.22.01533

[24] Jimenez RB , Schenkel C , Levit LA , et al. Oncologists' perspectives on individualizing dose selection for patients with metastatic cancer. JCO JOP. 2022;40:1531. doi:10.1200/OP.22.00427 36126244

[25] Schrag D , Hershman DL , Basch E . Oncology practice during the COVID‐19 pandemic. JAMA. 2020;323(20):2005‐2006. doi:10.1001/jama.2020.6236 32282023

[26] Cinar P , Cox J , Kamal A , et al. Oncology care delivery in the COVID‐19 pandemic: an opportunity to study innovations and outcomes. JCO Oncol Pract. 2020;16(8):431‐434. doi:10.1200/OP.20.00326 32463764

[27] Brunt AM , Haviland JS , Wheatley DA , et al. Hypofractionated breast radiotherapy for 1 week versus 3 weeks (FAST‐forward): 5‐year efficacy and late normal tissue effects results from a multicentre, non‐inferiority, randomised, phase 3 trial. Lancet. 2020;395:1613‐1626. 32580883 10.1016/S0140-6736(20)30932-6 PMC7262592

[28] Basch E , Abernethy AP . Supporting clinical practice decisions with real‐time patient‐reported outcomes. J Clin Oncol. 2011;29:954‐956. 21282536 10.1200/JCO.2010.33.2668

[29] Hsien S , Sussman J , Martelli‐Reid L , Pond G , Bainbridge D . Do high symptom scores trigger clinical actions? An audit after implementing electronic symptom screening. J Oncol Pract. 2012;8(6):e142‐e148. doi:10.1200/JOP.2011.000525 23598849 PMC3500488

[30] StratFrame. https://www.cancercareontario.ca/sites/ccocancercare/files/assets/CCOPatientOutcome

[31] Yim WW , Yetisgen M , Harris WP , Kwan SW . Natural language processing in oncology: a review. JAMA Oncol. 2016;2:797‐804. 27124593 10.1001/jamaoncol.2016.0213

[32] Kurian AW , Mitani A , Desai M , et al. Breast cancer treatment across health care systems. Linking electronic medical records and state registry data to enable outcomes research. Cancer. 2014;120:103‐111. 24101577 10.1002/cncr.28395 PMC3867595

[33] Karimi YH , Blayney DW , Kurian AW , et al. Development and use of natural language processing for identification of distant cancer recurrence and sites of distant recurrence using unstructured electronic health record data. JCO Clin Cancer Inform. 2021;5:469‐478. 33929889 10.1200/CCI.20.00165 PMC8462655

[34] Hernandez‐Boussard T , Blayney DW , Brooks JD . Leveraging digital data to inform and improve quality cancer care. Cancer Epidemiol Biomarkers Prev. 2020;29(4):816‐822. doi:10.1158/1055-9965.EPI-19-0873 32066619 PMC7195903

[35] Morin O , Vallières M , Braunstein S , et al. An artificial intelligence framework integrating longitudinal electronic health records with real‐world data enables continuous pan‐cancer prognostication. Nat Cancer. 2021;2:709‐722. doi:10.1038/s43018-021-00236-2 35121948

[36] Brierley J , Gospodarowicz M , Wittekind C , eds. TNM classification of malignant Tumours (8th edition). Union for International Cancer Control. New York: Wiley Blackwell; 2017.

[37] Unger‐Saldaña K . Challenges to the early diagnosis and treatment of breast cancer in developing countries. World J Clin Oncol. 2014;5(3):465‐477. doi:10.5306/wjco.v5.i3.465 25114860 PMC4127616

[38] Cooper JD , Shou K , Sunderland K , Pham K , Thornton JA , DeStefano CB . Real‐world pitfalls of analyzing real‐world data: a cautionary note and path forward. JCO Clin Cancer Inform. 2023;7:e2300097. doi:10.1200/CCI.23.00097 37729597 PMC10569773

