# Supplementary material for: Breast cancer learning health system: Patient information from a data and analytics platform characterizes care provided
Source: Learn Health Syst. 2024 Feb 13;8(3):e10409. doi: 10.1002/lrh2.10409 (PMC11257056; doi:10.1002/lrh2.10409)
Supplement: Supplementary file 1 — Data S1. Supporting information. [file LRH2-8-e10409-s001.docx]

**SUPPLEMENT ON-LINE**

**Table S1: Ontario Marginalization Index**

| The Ontario Marginalization Index (ON- Marg) is an area-based index that seeks to show differences in marginalization between geographic areas and understand inequalities in various measures of health and social well-being, either between population groups or between geographical areas. It has four dimensions: material deprivation, ethnic concentration, dependency, and residential instability. Each dimension of marginalization is a composite measure made up of several more granular indicators. Material deprivation is closely connected to poverty and includes income, quality of housing, education attainment and family structure characteristics. Ethnic concentration refers to the degree of area level density of people who are recent immigrants and/or people belonging to a visible minority. Dependency captures those who do not have income from employment. This includes seniors, children, and adults whose work is not financially compensated (adults included in this measure may be taking care of others or may be prevented from working due to disability). Residential instability captures neighborhood quality, cohesiveness, and support by measuring the types and density of residential accommodations as well as certain family structure characteristics.  Matheson FI, Moloney G, van Ingen T; Ontario Agency for Health Protection and Promotion (Public Health Ontario). 2016 Ontario marginalization index: user guide. 1st revision. Toronto, ON: St. Michael’s Hospital (Unity Health Toronto); 2022. Joint publication with Public Health Ontario. |
| --- |

**Table S2: Systemic Therapy Agents**

| **Regimen** | **Adjuvant** | **Neoadjuvant** | **Palliative** |
| --- | --- | --- | --- |
| AC | 122 | 8 | 17 |
| AC-DOCE | 8 | 17 | 0 |
| AC-PACL | 22 | 22 | 1 |
| AC-PACL(DD) | 934 | 676 | 2 |
| AC-PACL(DD)+TRAS | 1 | 0 | 0 |
| AC-PACL(W) | 72 | 24 | 0 |
| ANAS | 2037 | 71 | 101 |
| CAPE | 41 | 0 | 190 |
| CAPELAPA | 0 | 0 | 4 |
| CISP(RT-W) | 1 | 0 | 1 |
| CISPGEMC(W) | 0 | 0 | 16 |
| CMF(PO) | 18 | 0 | 2 |
| CRBP | 0 | 0 | 4 |
| CRBPDOCETRAS | 12 | 8 | 0 |
| CRBPGEMC | 0 | 0 | 11 |
| CRBPGEMC(W) | 0 | 0 | 12 |
| CRBPPACL | 0 | 0 | 2 |
| CYCL(PO) | 0 | 0 | 7 |
| CYCLDOCE | 108 | 6 | 1 |
| DOCE | 2 | 0 | 2 |
| DOCE(W) | 1 | 0 | 3 |
| DOCE+PERT+TRAS | 0 | 0 | 18 |
| DOCE+TRAS | 0 | 0 | 3 |
| DOXO(W) | 0 | 0 | 33 |
| ERIB | 0 | 0 | 35 |
| ETOP(PO) | 0 | 0 | 1 |
| EVEREXEM | 0 | 0 | 15 |
| EXEM | 126 | 5 | 39 |
| FAC | 0 | 2 | 2 |
| FEC-D | 5 | 0 | 0 |
| FLVS | 1 | 0 | 5 |
| FLVSPALB | 0 | 0 | 2 |
| GEMC | 0 | 0 | 5 |
| GOSE | 2 | 0 | 4 |
| KADC | 40 | 0 | 25 |
| LETR | 646 | 76 | 242 |
| LETRPALB | 2 | 2 | 153 |
| LETRRIBO | 0 | 0 | 12 |
| LPRL | 7 | 0 | 3 |
| MEGE | 0 | 0 | 4 |
| NPAC | 0 | 0 | 26 |
| PACL | 0 | 2 | 7 |
| PACL(W) | 3 | 2 | 141 |
| PACL(W)+TRAS | 5 | 1 | 0 |
| PACL+PERT+TRAS | 0 | 0 | 22 |
| PACL+TRAS | 0 | 0 | 4 |
| PEMB | 0 | 0 | 1 |
| PERT+TRAS | 1 | 0 | 71 |
| TMXF | 1211 | 25 | 104 |
| TRAS | 554 | 163 | 41 |
| TRAS(W) | 1 | 0 | 0 |
| VINO | 0 | 0 | 4 |
| XELOX | 1 | 0 | 0 |

A=doxorubicin, C=cyclophosphamide, DOCE=docetaxel, PACL=paclitaxel, ANAS=anastrozole, CAPE=capecitabine, LAPA=lapatinib, CISP=cisplatin, GEMC=gemcitabine, CRBP=carboplatin, EXEM=exemestane, ETOP=etoposide, ERIB=eribulin, EVER=everolimus, F=fluorouracil, FLVS=fulvestrant, GOSE=goserelin, KADC=kadcyla, LET=letrozole, LPRL= leuprorelin, M=methotrexate, MEGE-megace, NPAC=nab-paclitaxel, PALB=palbociclib, PERT=pertusumab,, RIBO=ribociclib, TRAS=trastusumab, VINO=vinorelbine, ZOLE=zoledex, TMXF=tamoxifen, XELOX=oxaliplatin and capecitabine

**Figure S1: Follow-Up**

**
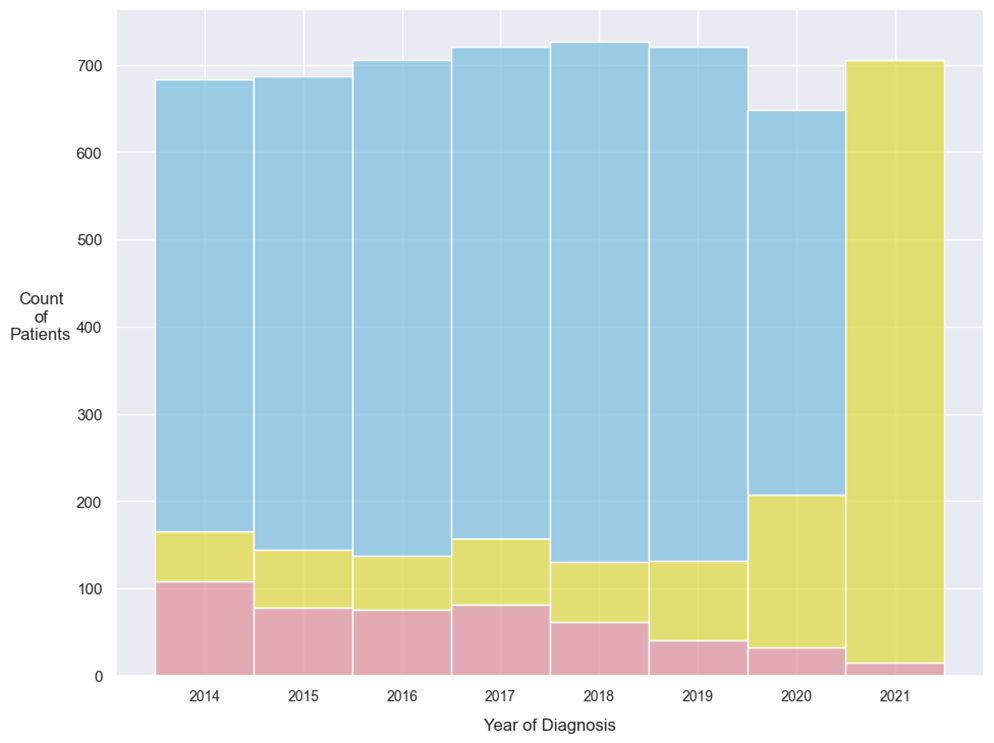
**

**Figure S1: Follow-up data available for patients by year of diagnosis**

Each bar illustrates the degree of follow up data available for patients diagnosed in a given year. The red portion of the bar illustrates the proportion of patients who have died, the yellow indicates the proportion for whom less than two years of follow up data is available, and the blue indicates the proportion for whom more than two years of follow up data is available. The proportion of patients with less than two years of follow-up is quite constant from 2014-2019 (mean 10%), with an expected increase in 2020 and 2021 (since two full years have not passed for many of these patients). Similarly, we observe an expected increase in the proportion of patients who have died in 2014 than for later years.

**Figure S2: ESAS in Patients on Adjuvant Chemotherapy**

**
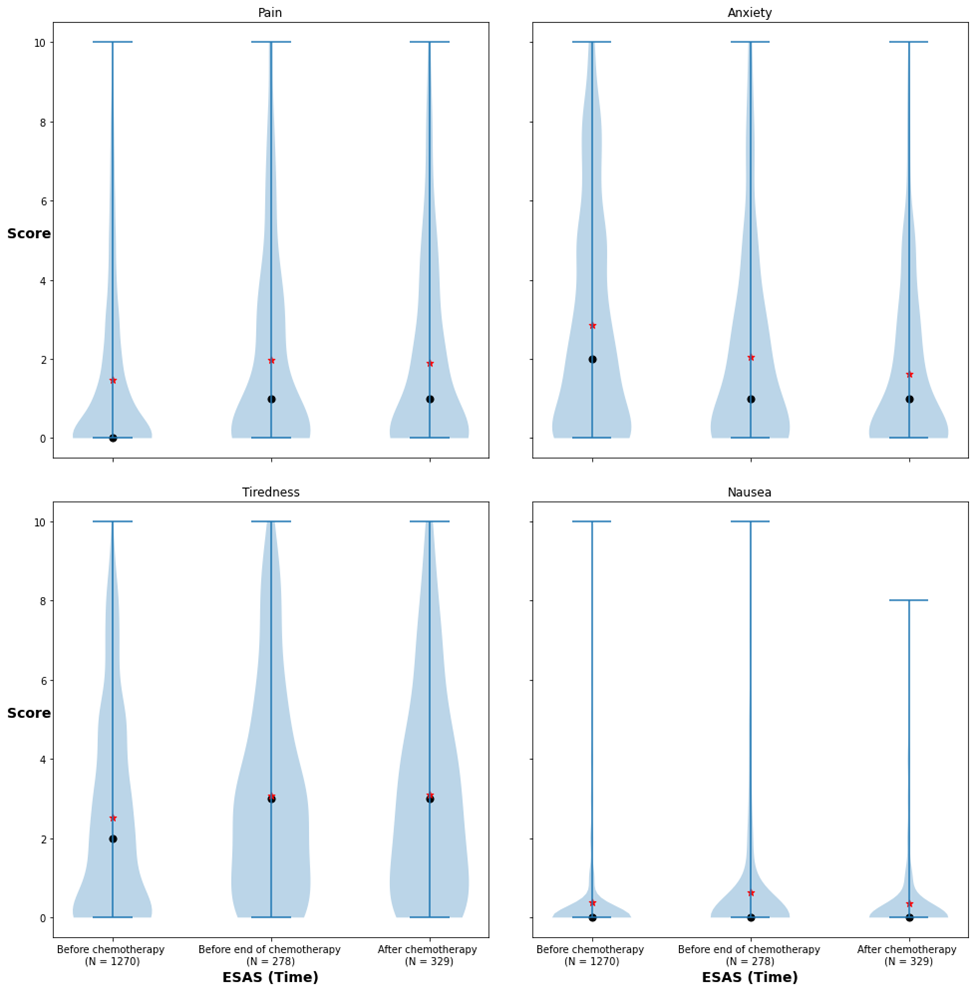
**

**Legend Figure S1**

The Edmonton Symptom Assessment Scale is a questionnaire used to rate the intensity of nine common symptoms experienced by cancer patients, including pain, tiredness, nausea, depression, anxiety, drowsiness, appetite, well-being and shortness of breath. Each question is scored by a categorical scale from 0 to 10, with 0 being no symptom and 10 being worst ever. A 1-point change on the scale is clinically important. In this figure, four symptoms are shown. Before chemotherapy is any ESAS completed within 4 weeks prior to starting chemotherapy. End of chemotherapy is within 4 weeks of completion and end of chemotherapy is within 4-12 weeks following last chemotherapy. Red dot is mean and black dot is median. The scores are for any patient who completed a questionnaire within a time window.

Hui D, Bruera E. The Edmonton Symptom Assessment System 25 Years Later: Past, Present, and Future Developments. J Pain Symptom Manage. 2017 Mar;53(3):630-643. doi: 10.1016/j.jpainsymman.2016.10.370. Epub 2016 Dec 29. PMID: 28042071; PMCID: PMC533717
